# Wearable device-based interventions in heat-exposed outdoor workers — a scoping review and an explanatory intervention model

**DOI:** 10.1186/s12889-025-24262-2

**Published:** 2025-08-22

**Authors:** Julian Friedrich, Teresa S. Schick, Filip Mess, Simon Blaschke

**Affiliations:** TUM School of Medicine and Health, Department of Health and Sports Sciences, Technical University of Munich, Munich, Germany

**Keywords:** Heat stress, Heat strain, Heat-related illness, Sensors, Devices, Health, Workplace, Environmental health literacy

## Abstract

**Background:**

Global climate change poses a challenge to the health prevention of heat-exposed outdoor workers. Interventions with mobile or wearable devices monitoring physiological and environmental parameters may be one solution to maintain and promote their health. Based on the recognized potential of wearables in mitigating heat stress, a detailed analysis of the contextual factors, mechanisms, and outcomes of wearable device-based interventions is lacking. A scoping review was carried out to address the objectives of contextual analysis, fundamental mechanisms, and an assessment of outcomes to propose an explanatory intervention model based on the findings.

**Methods:**

Web of Science and PubMed databases were searched by search strings related to (1) wearables (2), outdoor workers, and (3) heat stress. Study characteristics and relevant data regarding the context-mechanism-outcome configurations were extracted and analyzed.

**Results:**

Out of 410 articles detected, 19 publications were eligible for in-depth review. Wearables are well-accepted for the prevention of heat stress symptoms. By recording relevant indicators, i.e., heart rate and temperature, real-time health alerts can be issued as risk-based early warnings, and personalized feedback or recommendations towards behavior adaptation can be generated. A high risk of occupational heat stress was identified for construction, agriculture, and groundwork workers. Heat-exposed outdoor workers were mainly young to middle-aged males and often overweight or obese, with increased heart and breathing rates in hot work environments. Wearable device-based interventions are particularly effective if a mindset of safety culture is present in the workplace and environmental health literacy is promoted to increase heat risk awareness and willingness to change work health behavior.

**Conclusion:**

Based on these findings, we developed an explanatory intervention model. This model draws on well-established frameworks, theories, and models. It helps to identify, describe, and explain what works, for whom, and under what circumstances in the context of wearable usage in heat-exposed outdoor workers. Incorporating environmental health literacy and precision prevention in occupational health approaches with continuous monitoring of environmental and physiological parameters will allow for real-time, tailored feedback, leading to more effective heat stress prevention.

**Supplementary Information:**

The online version contains supplementary material available at 10.1186/s12889-025-24262-2.

## Background

Global climate change alters weather patterns, leading to increased extreme weather conditions, including rising temperatures and more frequent heat waves [1]. This trend is particularly concerning in the work context [2–4]. People typically spend around 40% of their waking hours at work [5]. Some workers spend more than 22% of their working time outdoors and are considered outdoor workers [6, 7] who are exposed to different climatic conditions. Over one billion workers worldwide experience high heat episodes, with approximately one-third suffering from adverse health effects [8]. These outdoor workers are increasingly at risk of heat stress. Occupational heat stress occurs when a worker engages in continuous physical activity in a hot environment [9]. Occupational heat stress manifests through symptoms such as excessive sweating, fatigue, tachycardia, hypotension, dizziness, and nausea at the workplace [10, 11]. This phenomenon poses significant health risks to outdoor workers. These include heat-related illnesses and increased physiological strain. In addition, it contributes to decreased labor productivity. It is also associated with a higher incidence of work-related injuries [12–14] and occupational heat-related deaths [15]. Productivity losses are closely linked to increases in heat stress. The Wet Bulb Globe Temperature (WBGT) is a heat stress index that accounts for temperature, humidity, wind speed, and solar radiation in direct sunlight [16, 17]. For each 1 °C rise in WBGT, global productivity is estimated to decline by nearly 10%. These productivity losses are projected to increase further, reaching 30 to 40% in the future [18].

Occupational heat stress is a major factor that negatively affects both workers’ health and labor productivity. It contributes to a substantial economic burden, both retrospectively and in future projections [19, 20]. As a result, the need for effective intervention strategies to prevent heat stress in outdoor occupations has been clearly identified [6]. However, the high variation of individual and contextual factors in the outdoor working environment represents a complex situation that poses a major challenge for effective tailored preventive interventions in occupational outdoor environments [21, 22]. Outdoor workers in different settings may experience fluctuating temperatures throughout the day. For example, emergency responders can be subject to unpredictable and extreme heat conditions [4]. Agricultural workers might face prolonged exposure to direct sunlight [23]. Construction workers deal with the added strain of heavy equipment and reflective surfaces that intensify heat [24, 25]. These diverse scenarios illustrate the multifaceted nature of managing heat stress, making it challenging to implement one-size-fits-all interventions [26, 27].

In response to these complex challenges for outdoor workers and the shortcomings of one-size-fits-all approaches, more innovative intervention strategies have been developed. Examples include portable shade structures [28] or targeted cooling interventions [29]. These measures aim to mitigate heat stress in specific populations of outdoor workers. One intervention strategy with a high potential for tailored heat stress prevention is wearable devices (wearables) to monitor and manage heat stress in outdoor work environments [30]. Wearables offer an effective solution to this complexity. They provide real-time, personalized data on both physiological and environmental conditions [31]. This enables immediate adjustments and more precise management of heat stress. Interventions can therefore be tailored to individual needs and varying environmental factors [22].

Wearables are tiny computer systems worn directly on the body or integrated into clothing [32]. Examples of wearables are smartwatches, smart glasses, headbands [32], wristbands [33], and ear canal thermometers [34]. Wearables offer continuous real-time monitoring capabilities [31]. They can sense, process, store, and transmit various types of data. These include physiological, psychological, environmental, and behavioral metrics [35]. This real-time data can inform timely interventions to prevent heat-related illnesses and optimize outdoor worker safety and performance [22, 30, 36]. Thus, wearables may serve as valuable new tools in the occupational prevention of heat stress and strain [33, 34]. Wearables are a particularly promising technology to protect outdoor workers’ health on an individual basis in hot working environments. They collect and extract useful, actionable health-related information on an individual basis. This allows for anticipating potential health issues caused by excessive physical demands [22, 37]. If, for instance, a wearable anticipates or detects an increase in body temperature, an alert can be issued. Simultaneously, protective measures like a notice to take a break in the shade may be communicated from a remote location or automatically to the user in real time to prevent adverse health effects. A recent scoping review provides an overview of available wearables to assess occupational heat stress risk [38]. Due to the article’s scope, the devices’ specifications, usage and validation, as well as test settings and conditions are provided. Furthermore, the authors expanded their search to occupational health outcomes. They concluded, among other findings from the current research, that wearables show great promise for monitoring heat stress, particularly in laboratory settings [38].

### Research gaps

Nevertheless, the scoping review by Cannady et al. [38] predominantly addressed the device-based technological perspective of using wearables to mitigate heat stress. It did not exclusively focus on the complexity of heat stress prevention in outdoor workers. In addition to important technological considerations related to wearable use for occupational heat stress prevention, practical knowledge remains limited. Specifically, there is a lack of understanding of how to effectively plan, implement and integrate wearable interventions for heat-exposed outdoor workers. This represents a critical gap in providing actionable guidance to optimize their real-world application and impact [38, 39]. To bridge this general research gap, it is essential to identify the contextual factors, key mechanisms, and intervention outcomes. This is crucial in clarifying the practical understanding of intervention feasibility and effects of using wearables in heat-exposed outdoor workers [40]. This theory-driven approach leads to an explanatory intervention model that consists of context-mechanism-outcome configurations to explain “what works, how, why, in which contexts, for whom, and to what extent” [40, 41]. Building on the recognized potential of wearables to mitigate heat stress and the need for an explanatory intervention model, three specific research gaps emerge. These gaps stem from the broader lack of guidance on effectively planning, implementing, and integrating wearable-based interventions.

First, there is a need for comprehensive intervention strategies explicitly tailored to outdoor workers, considering the high variability in individual responses and environmental conditions [12]. Thus, current literature often lacks a detailed analysis of the contextual factors that influence the effectiveness of wearable device-based interventions (e.g., work environment and preconditions of technology use). Second, there is limited information on the mechanisms (e.g., the assessment of heat stress indicators and wearable feedback) of how wearables can best be utilized to prevent heat stress and heat-related health issues [38]. For instance, knowledge of the monitored heat-related parameters, chosen thresholds, and the reported feedback for outdoor workers is currently rare. Third, intervention outcomes resulting from the respective contextual factors and mechanisms, such as the risk of heat illness and heat-related behavior or perceived usability of wearables, require further investigation. A recent study [42] claims a narrow set of physiological outcomes. A broader set of outcome parameters could help to identify which factors most effectively drive behavior change and enhance user engagement in heat stress prevention.

### Objectives

This scoping review aims to provide a comprehensive overview of wearable device-based interventions in heat-exposed outdoor workers. The objectives include the following context-mechanism-outcome configurations:

1. A contextual analysis: To identify the specific contexts in which wearable device-based interventions have been implemented for heat-exposed outdoor workers.
2. The key mechanisms: To investigate the critical mechanisms and features of wearable technologies that are relevant for managing heat stress among outdoor workers.
3. An outcomes assessment: To evaluate the outcomes of wearable device-based interventions in reducing heat-related illnesses and improving worker safety and health.

Furthermore, with the results of this scoping review, we aim to suggest an explanatory intervention model based on the findings, providing a model for future research and practical applications in the field of wearable usage in heat-exposed outdoor workers.

## Materials and methods

To explore the context-mechanism-outcome configurations, we conducted a scoping review following the Joanna Briggs Institute Reviewers’ Manual [43] and the Preferred Reporting Items for Systematic Reviews and Meta-Analyses Extension for Scoping Reviews (PRISMA-ScR) [44].

### Information sources

Literature searches were conducted in July 2024 in two online databases (Web of Science™ and PubMed^®^) that cover most scientific fields [45]. Additionally, pursuing references of references of included articles (i.e., forward and backward citation tracking) was conducted to identify previously undetected articles by the literature search.

### Search strategy

Based on a preliminary non-systematic feasibility search, database search strings for title and abstract search were developed with terms and synonyms related to three categories: (1) wearables, (2) outdoor workers, and (3) heat stress. The Boolean Operator OR connected the search terms of each group. The Boolean Operator AND connected search term groups (see Additional file 1). Searches focused on English literature with no restriction on the time until July 2024.

### Eligibility criteria

Except for reviews and meta-analyses, all types of publications were eligible. Articles identified through the search strategy were included only if the full text was available and they satisfied the eligibility criteria regarding population, types of interventions, context, outcomes, and types of evidence (see Table 1).

**Table 1:** Eligibility criteria

Category	Inclusion	Exclusion
Population	Adult outdoor workers, e.g., construction workers	All other workers, e.g., indoor or office workers
Types of intervention	Implementation of heat-related wearable device-based intervention or related survey to: - minimize the risk of accidents, - maintain or prevent health, - monitor health parameters.	No wearable device-based interventions
Context	Interventions delivered in heat-exposed outdoor workplaces Any geographical location	Indoor workplaces
Outcome	At least one health-related parameter monitored or discussed in a survey Heat illness-related outcomes Health prevention and health promotion	No health outcomes No information on the effectiveness of the intervention
Types of Evidence	Primary empirical research studies, e.g., randomized controlled trials, cohort studies, cross-sectional studies, case reports Protocols for planned studies Conference Proceedings Grey literature Letters to the editor and commentaries Articles written in English Full-text articles available	Reviews and meta-analyses Articles not written in English

### Screening

**Fig. 1:**
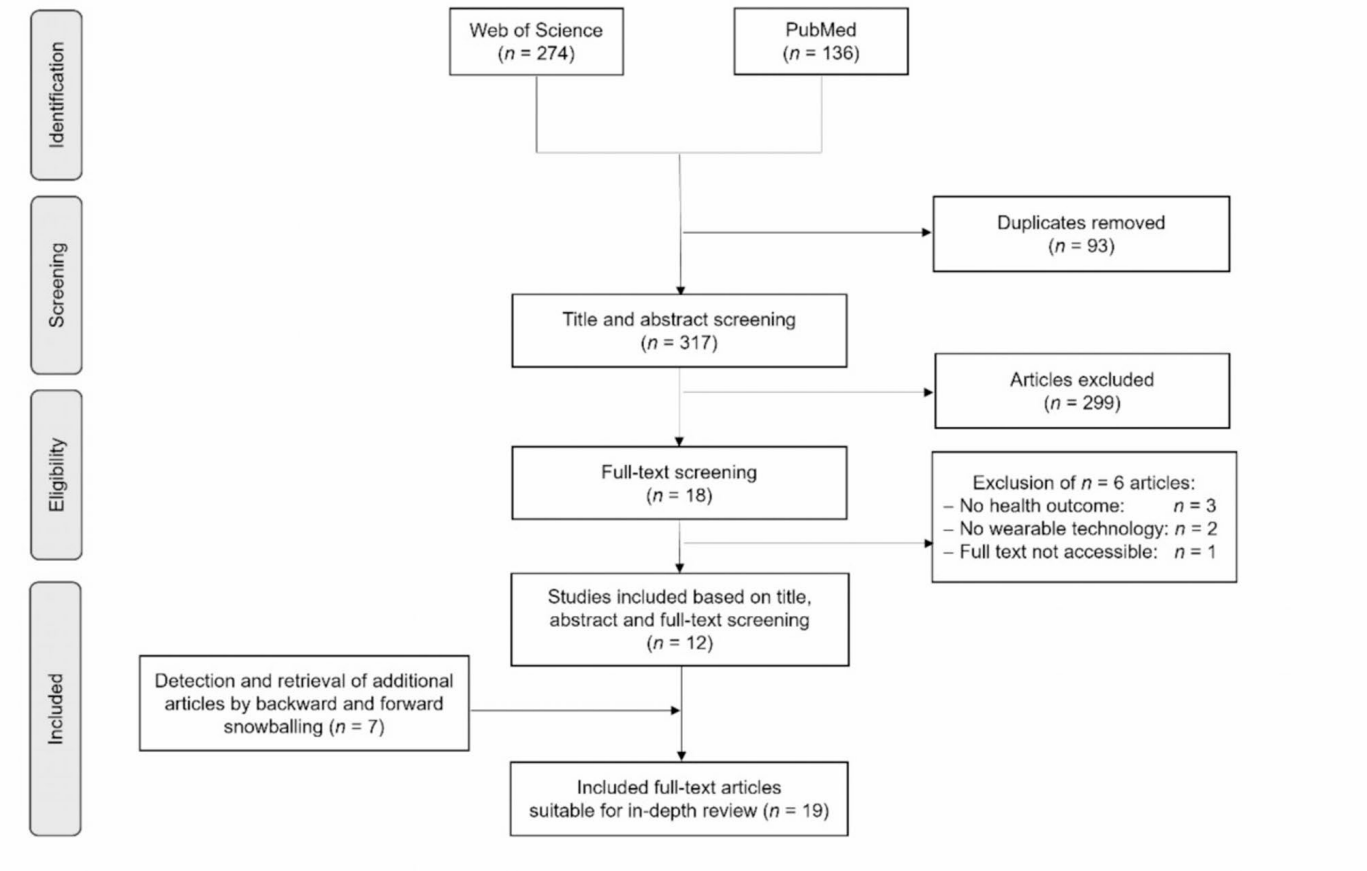
PRISMA-ScR flow chart representing search results along the screening process

All articles found were exported and transferred to the “Rayyan App” (http://rayyan.qcri.org) [Rayyan Systems, Boston, Massachusetts, USA] [46]. The “Rayyan App” is a free web-based collaboration platform for systematic reviews for publication screening. A total of 410 articles were found, and duplicates were removed. A screening of 317 articles by title and abstract was performed independently by two reviewers. Articles were excluded if they did not meet the eligibility criteria. The full texts of all papers selected for the scoping review were then retrieved to closely evaluate their introductions, methods, and results, to confirm whether the initial assessments made from their titles and abstracts were appropriate. Additionally, seven relevant articles meeting eligibility criteria and detected by citation tracking or a complementary purposive Google Scholar search were included. Finally, nineteen articles were left for in-depth review. The yield of the database search is schematically illustrated in Fig. 1.

### Data extraction, analysis, and synthesis

For data appraisal and analysis, general study characteristics (e.g., title, author, year, country of the first author/country in which the study was conducted, study purpose, methods, participant characteristics) and specific data matching the objectives of this scoping review (contexts-mechanisms-outcomes configurations) were extracted and transferred into a spreadsheet using Excel^®^ software [Microsoft Corporation, Redmond, Washington, USA]. Two reviewers formulated and discussed the themes of the different higher-order categories, contexts, mechanisms, and outcomes. Finally, data analysis was conducted by counting the different themes. Data was synthesized by summarizing the findings in a table created with Excel^®^ software [Microsoft Corporation, Redmond, Washington, USA].

Furthermore, we considered the relevance, richness, and rigor of the included studies in relation to their contribution to context-mechanism-outcome configurations. This approach is informed by realist review principles [47], acknowledging the value of studies based on their theoretical contribution rather than methodological quality alone. Relevance refers to how well a study contributes to building or testing the context-mechanisms-outcome configuration [47]. Richness highlighted the depth of conceptual explanation and contextual detail a study offers, helping determine its usefulness in understanding how and why an intervention works [48]. Rigor assessed the credibility and trustworthiness of the methods used to generate the findings, recognizing that even studies with weaker designs can provide valuable insights [47].

## Results

The following section summarizes the results of 19 scientific articles suitable for in-depth review with the contextual factors of the studies, mechanisms, and outcomes of interventions. All the included studies are listed in Additional File 2. The specific study details are in Additional file 3.

### Contextual factors of the studies

Four different contextual factors of wearable device-based interventions for heat-exposed outdoor workers emerged: (1) Study contexts and sample characteristics (2), physical and psychosocial work environments (3), health resources, and (4) preconditions of technology use.

#### Study contexts and sample characteristics

The 19 included articles were published between 2015 and 2023. Most of the studies (10 out of 19, or 53%) were published in the United States of America (USA) [37, 49–57], followed by three studies (16%) from Japan [58–60]. One study each came from Brazil [61], India [62], Mexico [9], the Netherlands [36], and the Republic of Korea [63]. Additionally, one study was conducted in Saudi Arabia [16], with the authors affiliated with the USA and Saudi Arabia. Detailed information on the countries included in each study can be found in Additional File 3.

The sample populations varied in size and characteristics: Sample sizes ranged from six individuals [51] to 588 individuals [52]. The average number of participants in the studies was 71, while the median was 36, indicating a skewed distribution due to a few large-sample studies. Five studies each focused on construction workers and farmworkers. Three studies included ground workers, and one study each focused on municipal workers, general landscape maintenance, custodial staff, and physically demanding work tasks. Two samples differ from the others, concentrating on students doing experimental construction work [53] or sand shoveling tasks in outdoor work environments [62]. We included these samples because of the real work situations, regardless of whether the participants are students carrying out this work as part of the study.

Based on the reported mean age and sex, outdoor workers were predominantly young to middle-aged male adults (*M* = 83% male). Five studies [16, 36, 54, 59, 62] included only male participants, and one study [58] concentrated only on female participants. Five studies reported “gender,” but only used binary categories (male/female) without clarifying whether this referred to sex or gender identity. No diverse identities were reported. The mean age of all study participants was 39 years, ranging from *Min* = 24 years to *Max* = 50 years.

The majority of ground workers in the study by Runkle et al. [37] were overweight with a Body Mass Index (BMI) between 25 and 30 kg/m² or obese (BMI > 30 kg/m²). Similarly, Culp & Tonelli [49] reported that about one-third, especially younger seasonal farmworkers, were obese or even morbidly obese (BMI > 40 kg/m²). Heat stress symptoms, such as extreme thirst, muscle cramps, feeling lightheaded or dizzy, were most often observed by workers with a BMI between 30 and 39 kg/m² [49]. Monitored heart and breathing rates were higher in overweight participants [49]. In contrast, a lower BMI was associated with a decreased risk of heat strain (i.e., a protective factor) [37].

#### Physical and psychosocial work environments

Thirteen of the 19 studies (68%) covered physical and/or psychosocial work environments. Some results of the included studies stated that the construction industry has the highest risk of occupational heat-related illness worldwide [59]. Work at construction sites is often characterized by restricted activities, including narrow scaffolding at high elevations, physically restricted spaces, or activities linked to a co-worker [16]. Therefore, work environments should allow construction workers to self-pace their work activities to prevent heat-related illnesses [16]. Furthermore, workers in the agricultural industry usually work under pressure to meet specific production quotas, thus being forced to work rapidly and have only limited breaks to rest [50]. Most farmworkers labor in fernery, which occurs in fields with limited ventilation and natural shade through trees [50]. In one study, most of the outdoor workers performing tasks related to landscaping, tree care, or ground maintenance reported removing their personal protective equipment due to the heat [37]. A health monitoring study across seasonal Hispanic farmworkers performing crop production tasks found that the main reason for not reporting symptoms of heat-related illnesses was the fear of not being rehired [49]. Therefore, the authors suggest that a psychosocial safety climate be established across farms, i.e., workers should not be viewed negatively if they report heat-related symptoms [49].

#### Health resources

Resources for the health of outdoor workers were addressed in three studies (16%). Construction workers were instructed to drink cool bottled water during their work activities, which the research team provided in unlimited amounts at the monitoring sites [16]. Employers of farmworkers ensured adequate fluid and caloric intake for high physical demands in hot environments, e.g., by providing water in the fields [49]. Additionally, fernery fields were covered by porous black shade cloth, and nursery workplaces were equipped with ventilators and fans [50]. Permanent outbuildings were established close to the workers, providing toilets and sinks [50]. Even an on-site clinic for heat-related illness symptoms was available in a particular setting [49].

#### Preconditions of technology use

Another contextual factor for wearables in outdoor workers is the preconditions of technology use addressed in three studies (16%). This component includes five preconditions for measuring and monitoring work exposures with sensor-based wearables in the workplace context [36]: (1) size and weight of sensor-based applications, (2) robustness of sensor-based technology applications, (3) time investment (e.g., workers should not spend time on data entry), (4) perceptibility of real-time feedback, and (5) company commitment and regulations. Furthermore, it is relevant to keep exposed users aware of the prevalent thermal work conditions at the individual level [62], which is possible with wearables. However, outdoor workers should understand the use of technology and be aware of its impact on their health. Thus, the introduction of wearables is as important as the interest of outdoor workers in sharing data with their doctors or health experts [55].

### Mechanisms of interventions

We identified three relevant underlying mechanisms for the effectiveness of wearable device-based interventions for heat-exposed outdoor workers: (1) determination of effective indicators for heat-related illness, (2) awareness of environmental hazards and worker involvement, and (3) feedback to users. For more detailed information regarding the mechanisms of each study, see Additional file 3.

#### Determination of effective indicators for heat-related illness

The definition of indicators for heat-related illness was identified in nine studies (47%) to define an additional component of mechanisms for evaluating and communicating outdoor workers’ current health status assessed by sensor-based wearables [50, 60]. Two different indicators exist based on measurements of heart rate or temperature.

The core body temperature has been reported to serve as a highly relevant measurable indicator to assess the development of heat stress [50, 52, 61, 62]. The World Health Organization (WHO) recommends that, during prolonged work in heat, core body temperature should not exceed 38 °C for workers in unmonitored settings. This threshold serves as a precautionary guideline, rather than a prescriptive limit value [50]. Other studies propose criteria to assess the risk of heat-related illness with a core temperature above 38.5 °C (101.3 °F) for at least one minute [52] or for acclimatized and medically selected individuals [61]. The threshold limit value for skin temperature is 43 °C (109.4 °F) [62]. Furthermore, occupational exposure limits based on Wet Bulb Globe Temperature (WBGT) measurements are used to assess and manage heat stress exposure in work settings. A critical threshold for WBGT is 33 °C (91.4 °F) [64].

The heart rate has been identified as another relevant parameter to detect the risk of heat-related illness in hot working environments, which can easily be monitored by sensor-based wearables [52, 59, 61]. Some authors mentioned that heart rate is determined by physical activity and endurance [61]. Therefore, a combination of heart rate monitoring and accelerometer data can estimate work intensity [52]. Nevertheless, the heat-related illness risk can be evaluated by measuring the maximum allowable heart rate (HR_max_), calculated by using the recommended equation: HR_max_ = 180 beats per minute (bpm) - age in years [50, 52, 61, 63]. In two other studies, a different formula based on the workplace determined by ISO9886:2004 [58, 59] is presented as follows: HR_max_ = 180 bpm − 0.65 x age in years. This formula applies a factor of 0.65 to represent the typical age-related decline in maximum heart rate, aiming to set a more conservative threshold that accounts for cardiovascular strain during work in the heat. Values above the threshold of one’s individual maximum heart rate monitored for several minutes indicate heat stress [52, 59, 61]. Based on these calculations, risk alerts can be issued to outdoor workers to prevent the progression of heat stress [52, 59, 61]. Again, heart rate and core body temperature are intertwined, while heart rate response is faster to changes in physical effort than core body temperature [61]. Therefore, work intensity should be included [52]. In another study, the heart rate of construction workers was monitored to assess the cardiovascular response [16]. The so-called heart rate reserve (HRR), a reliable measure of cardiovascular strain, was calculated [16]. Results can then be classified into three cardiovascular strain risk levels: low (HRR < 25%), moderate (HRR 25–29%), and high (HRR ≥ 30%) [16].

#### Awareness and involvement

Environmental hazard awareness was mentioned in eight studies (42%). A general lack of heat risk awareness was observed in a sample of ground workers [37]. Nevertheless, some outdoor workers were interested in tracking heat exposure to increase individual awareness [55]. An adequate perception of heat exposure as an occupational health hazard was associated with a lower risk of heat strain [37]. Thus, individual awareness of heat as an occupational hazard can be protective against developing heat strain at work [37].

Furthermore, two studies (11%) explicitly mentioned worker involvement. Perceptions and attitudes toward using digital technology are important and show the workers’ involvement and willingness to wear a technical device and participate in future studies on using sensor-based wearables. For example, during the recruitment phase, interested farmworkers came to the field sites to learn about the study [50], or ground workers were given examples of different sensor-based wearable devices and the opportunity to ask questions [55]. Therefore, it is necessary to find an appropriate and comprehensible way to communicate wearable-based results to outdoor workers [36, 37, 50, 55, 62]. This leads to an increased understanding and awareness of the individual heat exposure risk, risk for heat-related illnesses, and the impact of work activities on workers’ health, and advises them to take preventive countermeasures.

#### Feedback

Two ways to communicate wearable-recorded results to outdoor workers could be identified in five studies (26%). The two ways were: (1) real-time feedback via health alerts or notifications [9, 55, 60, 61] and (2) real-time report-back visualizations of collected health data [36]. Most of the participants would change their health behavior at work in response to notifications of excessive heat (84%) [55] or personalized heat prevention recommendations based on individual characteristics, for example, tasks, height, or weight (76%) [55]. Feedback on health-critical exposure levels by tactile and visual reaction would help workers directly promote their health and preventive behavior at work [36]. In a heat alert system, workers at risk were identified by a wearable monitoring device based on two relevant indicators of poor physical condition, i.e., heart rate and core temperature increase [60]. Workers who received a health risk alert via smartphone application were then reminded to rest to avoid symptoms of heat-related illness [60]. Simultaneously, heat alerts were also sent to the remote monitoring systems of the supervisors, who were then able to instruct the workers at risk to take a break [60].

### Outcomes of interventions

Three outcomes of wearable device-based interventions for heat-exposed outdoor workers were identified: (1) healthy workplaces, (2) action and adoption of health-promoting behavior, and (3) technology use. For further information on the outcomes of each study, see Additional file 3.

#### Healthy workplaces

All included studies aimed to support a healthy workplace by detecting the risk of developing heat-related illness [16, 49, 52, 58, 59, 61] and preventing heat stress-induced symptoms [54, 60]. Seventeen out of the 19 studies explored sensor-based wearables in outdoor workers by measuring and monitoring at least one of the following parameters: individually experienced temperatures, core body temperatures, ambient temperatures (*n* = 16 studies), heart rate (*n* = 15 studies), and physical activity level (*n* = 5 studies). Categories of monitoring and physiological or environmental biometrics are summarized in Table 2. Furthermore, there were isolated studies on subjective experiences of heat stress and strain [53] or the survey of the psychological comfort level [63]. Knowledge, attitudes, and practices regarding extreme heat were also surveyed [56]. In two cases, health promotion [36] or health and workplace safety [55] were addressed at a more organizational level.

**Table 2:** Summary of the included studies with study design, categories of monitoring and physiological or environmental biometrics

Authors and years	Study design	Categories of monitoring	Biometrics
Al-Bouwarthan et al., 2020 [16]	Field study, observational	physiological monitoring; physical activity monitoring	heart rate; physical activity
Culp & Tonelli, 2019 [49]	Descriptive observational study	multi-parameter monitoring	heart rate; physical intensity
Hertzberg et al., 2017 [50]	Methodological/analytical study	multi-parameter monitoring	heart rate; temperature; physical activity
Kakamu et al., 2021 [58]	Field study, cross-sectional	physiological monitoring; temperature monitoring; environmental monitoring	heart rate; skin temperature; humidity; wet bulb globe temperature
Kakamu et al., 2022 [59]	Field study	multi-parameter monitoring	heart rate; skin temperature; kilocalories per hour
Kim, A. & Yoo, 2023 [63]	Sensor-based field risk assessment	physiological monitoring; temperature monitoring; psychological comfort survey	heart rate; temperature; psychological comfort level
Kim, Y.-S. et al., 2022 [51]	Device development and pilot study	multi-parameter monitoring	heart rate; skin temperature, electrocardiograms, physical activity
Mitchell et al., 2017 [52]	Observational cohort study	multi-parameter monitoring	heart rate; temperature; physical activity
Pancardo et al., 2015 [9]	System design and validation	physiological monitoring; temperature monitoring	heart rate; humidity; temperature
Ruas et al., 2020 [61]	Observational study	physiological monitoring; temperature monitoring	heart rate; temperature
Runkle et al., 2019 [37]	Field validation study	physiological monitoring; temperature monitoring	heart rate; experienced temperature; daily heat symptoms
Shakerian et al., 2021 [53]	Sensor-based case study	physiological monitoring; temperature monitoring	heart rate; electrodermal activity; temperature
Sharma et al., 2022 [62]	Wearable-based observational study	physiological monitoring; temperature monitoring; environmental monitoring	heart rate; skin temperature; dry-bulb temperature; wet-bulb temperature; globe temperature; relative humidity
Spook et al., 2019 [36]	Needs assessment (qualitative)	interviews on wearable sensor technology	-
Sugg et al., 2018 [54]	Observational (sensor-based) study	physiological monitoring; temperature monitoring	heart rate; experienced temperatures
Sugg et al., 2020 [55]	Qualitative interview study	survey on digital devices	-
Togo & Hirata, 2021 [60]	System design and prototype demo	physiological monitoring; temperature monitoring	heart rate; temperature/humidity inside clothes
Uejio et al., 2018 [56]	Observational study	environmental monitoring	environmental temperature; humidity
Wang et al., 2019 [57]	Personal sampling field study	environmental monitoring	environmental temperature: wet bulb globe temperature

Multi-parameter monitoring includes physiological, temperature, and physical activity monitoring

A study on outdoor construction workers identified 12% of workers at high risk for heat-related illness [59]. A high cardiovascular strain was observed in construction workers on 79% of the days when plasterers engaged in restricted activities [16]. Individual temperatures vary broadly among ground workers at the same outdoor work location. In contrast, nearly 90% of ground workers found such information on personal ambient temperature helpful in mitigating their heat exposure at work [54]. Therefore, monitoring the heat stress of outdoor workers is crucial for preventing heat-related illness and promoting a healthy workplace. Identifying that many workers are at high risk for heat-related conditions [59] highlights the importance of early detection and targeted interventions [54]. The high cardiovascular strain observed emphasizes the need for adjusting workloads and implementing protective measures [16].

#### Action and adoption of health-promoting behavior

Action and adoption of health-promoting behaviors were identified in five studies (26%). The underlying mechanisms of feedback prompt behavioral changes and result in the adoption of preventive measures to promote the health of outdoor workers [55, 60]. For example, workers receiving the heat alert paid attention to their own physical conditions and proactively kept out of the heat to rest before becoming uncomfortable [60]. Simultaneously, according to the alerts, their supervisors assessed workers’ physical conditions and advised them to take a break. Since no severe outcomes, such as heat strokes, occurred during the study phase, the authors consider this health risk alert system useful [60]. Furthermore, the most prevalent work-related heat adaptation was drinking plenty of fluids [56, 62] or wearing a hat or light/loose-fitting clothing to cope with heat exposure [56]. Younger workers (83%) were more willing to receive personalized heat prevention recommendations and change behavior than older workers (67%) [55]. Furthermore, if possible, maintaining work-rest periods [60, 62], rescheduling heavy work activities to cooler times of the day [56], self-pacing [16], or job rotation [62] are useful ways of adapting the working day to the heat for outdoor workers.

#### Technology use

A highly relevant enabler for the effective use of wearable-based technology, identified in five studies (26%), is technology acceptance, for example, of procedures and equipment [52]. Outdoor workers expressed a preference for wearable sensor technology applications [36] with a smartphone app for health risk alerts or visualizations [60] instead of ingestible devices like heat pills, which transmit the body temperature to a sensor [50]. Participants were most likely to want frequent health monitoring, like blood pressure (63%) and heart rate (63%), often and all the time combined, for environmental monitoring like heat (72%) and workplace safety (47%) [55]. Furthermore, in one study, measuring and monitoring work exposures, data privacy/ownership, and positive feedback were reported to facilitate motivation for prolonged use of sensor technology applications [36]. If the workers receive real-time feedback on exposures (e.g., physical job demands, occupational heat stress, or noise) positively, and the workers can decide on sharing the data with general practitioners in the company, the long-term use of sensor technology increases [36]. If there are concerns regarding data privacy or the data is being used against the workers, they may disclose their data, which poses a barrier to the further use of a wearable [36, 55]. It is, therefore, essential that data protection is observed and communicated to employees and that their needs are considered to win them over in the long term [55].

#### Evidence base and contribution to the explanatory intervention model

To provide a clearer picture of the evidence base, we assessed the included studies according to their relevance, richness, and rigor as suggested by realist review principles [47]. Additional file 4 qualitatively evaluates each study’s contribution to the context-mechanism-outcome configurations. This appraisal helps illustrate the strength and depth of evidence underpinning the explanatory intervention model.

## Discussion

The aims of this study were (1) understanding the contextual factors influencing health outcomes in heat-exposed outdoor workers, (2) identifying key mechanisms and linking these factors with (3) wearable device-based intervention outcomes. Based on these results, we aimed to propose an explanatory intervention model for wearable usage in heat-exposed outdoor workers. A scoping review was conducted to identify relevant studies on heat-exposed outdoor workers. Of the 410 studies initially screened, 19 studies were selected for an in-depth review. The different studies showed a diverse overview of wearable device-based interventions for heat-exposed outdoor workers with different methodological approaches, including all aspects of context-mechanism-outcome configurations.

### Contextual factors

Several contextual factors emerged from the scoping review that contribute to the health risks of heat-exposed outdoor workers. Most studies primarily focused on male workers from labor-intensive industries like construction, agriculture, and groundwork. These outdoor workers often face compounded risks due to obesity, pre-existing health conditions, and a lack of sufficient health and safety measures on the job [37, 49]. These factors exacerbate the health risks associated with heat exposure [65]. The physiological strain imposed by working in high temperatures can lead to conditions such as heat stress, dehydration, heat exhaustion, and even heat stroke [66]. The most significant contributors to heat-related health issues are extreme temperatures in the work environments, combined with long working hours and strenuous physical tasks [12]. Outdoor workers, for example, farmworkers, often have limited access to cooling systems, shaded areas, and adequate hydration, exacerbating their vulnerability to heat illnesses [16, 50]. In addition to the inherent dangers of these environments, the limited access to health resources and the physical preconditions of the outdoor workers make it critical to explore effective intervention strategies emphasizing the need for tailored interventions in these vulnerable populations.

Pre-existing health conditions like obesity were highlighted as critical factors that make certain workers more prone to heat-related illnesses [37]. Thus, these individuals will benefit even more from wearable device-based interventions [49]. Regarding a previously reported high prevalence of obesity among construction workers (28%) in the USA [67–69] and overweight or obese farmworkers (81% of male workers and 76% of female workers) [70], this appears to be of particular relevance.

All the studies included were published in or after 2015. On the one hand, the introduction of wearable technology started around this time, for example, with personal activity trackers and the first Apple Watch in 2014 [71]. On the other hand, there was a growing interest in the effects of rising global temperatures, extreme weather, and climate change on worker health [72, 73]. Regarding regional differences, more than half of the studies were conducted in the USA. Almost one-third of the citizens in the USA used a wearable device in 2020 [74]. This underlines the higher establishment and distribution of wearables in high-income countries [75]. Furthermore, the adoption of health-monitoring technologies, such as wearable devices to measure heart rate and core body temperature, has been suggested to monitor worker health in real time. Nevertheless, the assessment of different intertwined biometrics is complex, and the devices were unreliable and less functional initially [76]. In a recent scoping review, the reliability and validity of wearables to monitor heat stress and strain were synthesized [38]. The authors reported an overestimation of temperature due to direct sunlight, overheating of the systems, and an all-beats detection failure or data loss due to movement, pressure, or not wearing the device tight enough [38, 77]. Wearables have become better at recording data over time. Nevertheless, direct validation or reliability testing of the devices in the outdoor work context is scarce [38]. Furthermore, considering contextual factors and mechanisms is becoming even more important in capturing usage and monitoring under certain circumstances. In accordance with the results of this scoping review, practical concerns such as the cost of these technologies, worker acceptance and compliance, and employer support were identified as barriers to widespread implementation [78–80].

### Mechanisms

Worker involvement and awareness of environmental hazards were substantiated as underlying mechanisms of wearable device-based interventions. Workers should be actively involved in such programs, and their opinions and ideas should be collected, listened to, and implemented [81]. The high usefulness of monitored results was identified by surveys and interviews on workers’ feedback, needs, and perceptions of wearable technology [36, 55].

Moreover, the connection between hazard awareness and environmental health literacy was identified. Environmental health literacy, which refers to an individual’s ability to understand, evaluate, and use environmental health information to reduce risk and improve both personal and environmental health [82], is essential in creating safer work environments. The use of wearable sensors to report individually collected data, such as heat exposure and heart rate, has shown promise in improving environmental health literacy among outdoor workers. For instance, when ground maintenance workers were equipped with wearable devices, the graphically visualized temperature and heart rate data helped 94% of participants better understand the health risks associated with heat exposure [82]. To ensure the effectiveness of interventions, occupational health professionals must develop tailored strategies that address the unique challenges faced by workers with low environmental health literacy, language barriers, or inadequate safety measures [49] and consider the complexities of their work environments, such as varying climate conditions and the physical demands of labor [83].

Feedback to users and the determination of indicators of heat-related illness, including physiological measurements like heart rate and core body temperature, were identified as two additional key mechanisms for effective interventions. Feedback to users (e.g., real-time report-back visualizations) appeared to be a powerful mechanism to improve environmental health literacy and promote wearable device-based environmental monitoring [82]. Moreover, the value of a health parameter report-back, together with comparative benchmarks and an interpretative context, has been reported for a public health setting and described as helpful in raising environmental health literacy on environmental exposures [84, 85].

### Outcomes

Three outcomes of wearable device-based interventions in outdoor workers could be identified: Healthy workplaces, action and adoption of health-promoting behavior, and technology use. The reviewed studies examined physiological outcomes such as core body temperature and heart rate in response to heat exposure. In a few studies, combined monitoring was performed using a multi-parameter monitoring wearable sensor measuring heart rate, breathing rate, skin temperature, and activity level [49]. Due to the correlation between heart rate and activity level, a combined evaluation is advisable. However, when using multi-parameter monitoring, it is important to consider how many parameters are recorded and ultimately reported back to the workers. An information overload can lead to excessive demands in already complex work situations [86]. However, these multi-component measurements reflect a holistic picture of the heat-exposed situation, which is currently poorly developed or lacking in the workplace [87].

Regarding technology use, data privacy is a consideration when deploying wearable sensors to monitor health outcomes. As these devices collect sensitive health information, there are risks related to unauthorized access and misuse of personal data. Ensuring that data is collected, stored, and processed in compliance with data privacy laws and regulations such as the General Data Protection Regulation is essential. Organizations must implement robust data protection measures and maintain transparency with users about how their data will be used and shared [88].

Despite the concerns regarding data privacy, there has been positive feedback regarding using multi-parameter monitoring wearable sensors. Users often report increased awareness of their physiological states, which can lead to better self-regulation and proactive measures to mitigate heat-related risks. For example, knowing their heart rate and body temperature can prompt users to take breaks, hydrate, or seek cooler environments, ultimately enhancing their safety and well-being [50, 60]. Several studies indicate that when users feel empowered by technology to manage their health, their overall satisfaction and engagement with the devices increase [89–91]. This feedback loop can foster a workplace culture of health and safety, encouraging employees to utilize these tools actively.

### Developing the explanatory intervention model

Pre-existing and well-established models, frameworks, or theories were selected and discussed in an iterative process to design an explanatory intervention model [40, 92]. To address the mentioned context-mechanism-outcome configurations [40] of the research questions, the following three models were selected for the present study to cover (1) policy and practices for improving workplace health to capture the contextual factors (2), health promotion and natural hazards preparedness to address the mechanisms and outcomes in using wearables, and (3) technology acceptance and usage behavior to capture both contextual factors and the outcomes of wearables in heat-exposed outdoor workers:

1. The WHO Healthy Workplace Model [81],
2. The Precaution Adoption Process Model [93, 94],
3. The Unified Theory of Acceptance and Use of Technology [95].

In the following, these three models or theories are briefly explained to give a more detailed insight.

#### The WHO healthy workplace model

The WHO Healthy Workplace Model was developed to provide a holistic and flexible framework for creating healthy workplace programs in various countries, workplaces, and cultures [81]. The WHO Healthy Workplace Model is comprised of four large “avenues of influence” (i.e., the content of issues), contains a process of continuous improvement, and is guided by the two core principles of leadership and worker involvement [81]. The four “avenues of influence” have been designated: (1) physical work environment, (2) psychosocial work environment, (3) personal health resources, and (4) enterprise involvement in the community [81]. They represent specific areas employers and workers can influence to create a workplace that protects and promotes all workers’ health, safety, and well-being [81]. In the present scoping review, the first three “avenues of influence” were considered particularly relevant as plausible contextual factors and mechanisms for the explanatory intervention model:

1. The physical work environment includes factors that may affect workers’ physical safety, (mental) health, and well-being, for example, physical hazards such as excessive heat. Means and ways to positively influence the physical work environment include, for instance, personal protective equipment, such as safety boots for construction workers, and training workers on safety procedures [81].
2. The psychosocial work environment refers to organizational culture, attitudes, values, or daily practices that can affect workers’ mental and physical well-being. Workers may experience psychosocial hazards as mental stressors, for example, problems with work demands and time pressures [81]. Ways to influence these psychosocial hazards could be the reduction of workload by reallocation of work or flexibility in the timing or location of work [81].
3. Personal health resources in the workplace are health services, information, or resources that aim to promote workers’ physical and mental health. Employers may enhance these personal health resources, for example, by providing medical services, including health assessments or medical surveillance [81]. Worker involvement deserves special attention due to its dependence on individual acceptance of and participation in effective, healthy workplace programs [81]. In the process of selecting and implementing wearable technology, worker involvement is likely to promote a positive user experience [96] and appears critical to the successful implementation of the new technology [97].

#### The precaution adoption process model

The Precaution Adoption Process Model [93, 94] is a health behavior model and well-established health promotion theory [98] and explains how individuals come to take action to prevent illness, injuries, or harm caused by external hazards and health threats [94, 98]. In this model, the process of adopting a new precaution or health-promotion behavior involves seven stages in which an individual may be: (1) unaware of potential health risks (2), unengaged (3), undecided about acting (4), decide not to act (5), decide to act by adopting a new precaution or behavior (6), act (7), maintain the protective behavior over time to mitigate health risks [94, 98]. The Precaution Adoption Process Model has previously been used to characterize technology adoption and describe behavioral responses to protective technologies in mine workers [98]. In that study, the Precaution Adoption Process Model has proven suitable for identifying barriers to technology acceptance and adopting protective behavior [98]. Therefore, the Precaution Adoption Process Model contributes important aspects of technology adoption to the explanatory intervention model of the present scoping review. In this context, unawareness (stage 1) of a health risk or necessary precaution behavior refers to heat stress and respective preventive measures. The transition from stage 1 “unaware” to one of the “decision-making” stages 2 to 7 may be supported by media messages specifically tailored towards hazards and precaution measures, but also by communication with significant others or personal experience of hazards [94]. In the context of wearable device-based interventions, stage 5) “decide to act” refers to the intention to use wearables as a preventive strategy against hazardous heat effects, while stage 6) “act” involves the actual use of wearables to support and guide preventive behaviors. If such behavior were repeated customarily, stage 7) “maintenance” would be achieved [94]. Outcomes of an intervention program depend on the various individual stages of program participants and may, therefore, vary accordingly regarding knowledge, skills, engagement, and readiness for healthy behavior [99, 100].

#### The unified theory of acceptance and use of technology

The Unified Theory of Acceptance and Use of Technology [95] integrates elements from eight popular user acceptance models. It addresses the acceptance of technology as a precondition for the use of technology and thus serves as a well-established theory to explain and predict technology use [95]. In the Unified Theory of Acceptance and Use of Technology, the behavioral intention to use new technology, i.e., wearable technology, is determined by the following preconditions: (1) performance expectancy, (2) effort expectancy, and (3) social influence. These preconditions for technology use determine both user acceptance and usage behavior of technology. Furthermore, the Unified Theory of Acceptance and Use of Technology also incorporates age, gender, experience, and voluntariness of use as four moderating variables that affect the influence of the direct determinants of technology use behavior [95]. In the original study by Venkatesh et al. [95], a diverse population across various sectors, including healthcare, education, and business environments, was involved. This broad demographic allowed for a comprehensive analysis of how user characteristics interact with the model’s constructs, providing insights applicable to a wide range of technology adoption scenarios [95]. For instance, younger users may have different expectations regarding technology performance than older users, while gender may influence perceptions of the effort required to use technology effectively [95].

### The explanatory intervention model

**Fig. 2:**
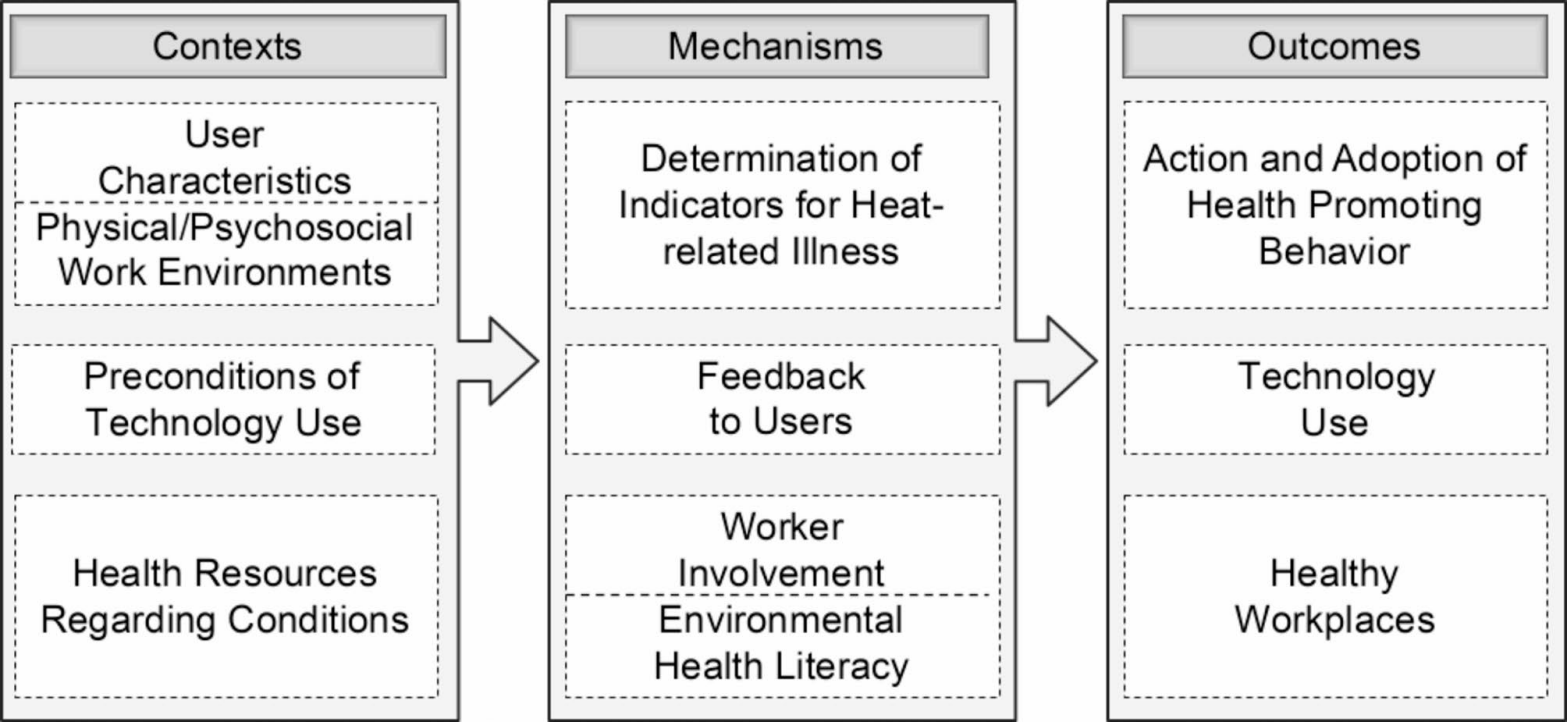
Explanatory intervention model (own illustration)

The explanatory intervention model (see Fig. 2) was developed based on the context-mechanism-outcome configurations identified in the scoping review. The included studies varied in their contribution to the context-mechanism-outcome configurations. Some offered rich contextual detail and plausible causal mechanisms (e.g., feedback systems, worker awareness), while others provided more limited or indirect insights. We considered this variation in the development of our intervention model and highlighted where assumptions were grounded in stronger theoretical contributions.

Contextual factors such as user characteristics, work environments, or preconditions of technology use were directly mapped to corresponding components in the model. Key mechanisms – like worker involvement or real-time feedback – emerged across the studies and were incorporated as central elements driving behavioral change and technology adoption. The model’s outcomes – behavioral adaptation, effective technology use, and healthy workplaces – reflect the aggregated findings of the reviewed interventions. Thus, the model synthesizes empirical insights from the literature into a structured framework for planning, implementing, and evaluating wearable device-based interventions for heat-exposed outdoor workers. Furthermore,

the various components extracted from the three mentioned models or theories enhanced the explanatory intervention model. Regarding contextual factors, (1) the aspects of user characteristics (e.g., age, gender, experience, and voluntariness of use), and (2) the preconditions of technology use (e.g., the size, weight, and robustness of sensor-based technology applications, the time investment and perceptibility of real-time feedback, and company commitment and regulations) were taken from the Unified Theory of Acceptance and Use of Technology. Additionally, the “avenues of influence” [81] were considered particularly relevant as plausible contextual factors for the explanatory intervention model to examine the effectiveness of wearable device-based interventions in outdoor workers, therefore, (3) the physical and psychosocial work environments and (4) personal health resources were found as results in the present scoping review and taken as influencing factors from the WHO Healthy Workplace Model. Most studies addressed specific work environments (e.g., construction, agriculture, and groundwork) as an essential influence on effective wearable device-based interventions. They added the psychosocial climate of safety while using a wearable and reporting symptoms of heat-related illnesses. This is accompanied by the health resources and conditions at work, such as the availability of shade and drinking water in the fields [49].

To address key mechanisms, the following aspects were included: (1) the determination of indicators for heat-related illness, (2) feedback to users, (3) worker involvement (from the WHO Healthy Workplace Model), and (4) environmental health literacy with its component awareness of environmental hazards (from the Precaution Adoption Process Model). The definitions of indicators and the heat-related illness risk measured by temperature and heart rate are important mechanisms in monitoring and reporting reliable and valid parameters. These parameters are linked with user feedback (e.g., report-back packets of heart rate and temperature). Most workers perceived the report-back of monitored temperatures and heart rates as very useful and thus were willing to change their protective behavior [91]. Therefore, worker awareness of environmental hazards and environmental health literacy are combined in the explanatory model. Together with worker involvement (e.g., perceptions and attitudes toward using digital technology), they can be a protective mechanism in developing heat strain at work [37].

Finally, the following outcomes were added: (1) the action and adoption of health-promoting behavior (from the Precaution Adoption Process Model), (2) technology use (from the Unified Theory of Acceptance and Use of Technology), and (3) healthy workplaces (from the WHO Healthy Workplace Model). Adopting health-promoting behaviors captures workers’ transition from awareness to actively implementing protective measures, such as staying hydrated or seeking shade. Technology use focuses on factors like ease of use, perceived usefulness, and trust in wearables, which are critical for ensuring consistent and effective device adoption. Together, these behaviors and technologies contribute to creating healthy workplaces where organizational culture and worker engagement foster sustained improvements in safety and well-being.

The spatial proximity of the factors in the model reflects their proximity in terms of content. In addition, the respective factors that are more likely to be related can be recognized on a horizontal visual axis. Exemplarily, physical, organizational, and social contextual factors include user characteristics and work environments under which outdoor workers operate. Indicators for heat-related illness serve as measurable mechanisms to identify when workers with specific user characteristics in a specific work environment are at risk of heat-related illness or when preventative actions should be triggered. For example, in a conducive work environment (e.g., a farming site or groundwork setting that offers shaded rest areas, portable cooling systems, or supportive policies like flexible work hours), workers with specific user characteristics (e.g., physical fitness levels, medical conditions such as heat sensitivity, or occupational roles like field laborers or machinery operators) wear advanced sensors that monitor their biometric data (e.g., body temperature, heart rate, physical activity, and hydration levels). These sensors provide real-time feedback on physiological metrics, visualizing the current status through clear indicators (e.g., green for safety, yellow for caution, and red for critical) and encouraging workers to adopt health-promoting behaviors, such as taking breaks, drinking water, or relocating to shaded areas. Additionally, based on this data, supervisors can dynamically adjust workloads, reassign tasks to cooler locations, or shift work hours to avoid peak heat, ensuring safety and optimizing productivity in physically demanding outdoor environments. Over time, these measures become ingrained as standard practices, reducing heat-related illnesses and fostering a culture of health promotion.

### Strengths and limitations

One of the primary strengths of this scoping review is that it broadens the usability of findings by expanding the scope to include the context-mechanism-outcome configurations [40]. While Cannady et al. [38] focus in a recent scoping review on identifying relevant devices and their reliability and validity, we synthesize the integration of the context-mechanism-outcome configurations that enhance the adaptability of insights across different contexts. The two reviews are a perfect complement to finding out which wearables can be used for heat-exposed outdoor workers [38] and to understand the respective mechanisms and rationale of how and why specific mechanisms lead to certain outcomes in certain contexts. This ensures that our findings are not limited to a particular domain or scenario, making the model more versatile and applicable to a broader range of settings.

Another strength review is the proposal of an explanatory intervention model that provides insights at different levels. This approach offers a model that can be useful for understanding and planning complex wearable device-based interventions for heat-exposed outdoor workers. By incorporating various dimensions, the model allows for more nuanced strategies in real-world applications, enhancing its practical relevance.

However, several limitations must be acknowledged. First, while a scoping review was conducted to inform the model development, it was not entirely systematic. This methodological choice was made to provide a broad overview of the available evidence. Still, it may have resulted in the omission of some relevant studies, limiting the comprehensiveness of the review. Consequently, while the scoping review provides valuable insights, future work would benefit from a fully systematic review with clear research questions to ensure that all relevant literature is considered.

Second, the subjective interpretation of findings presents a potential source of bias. Although we have taken measures to ensure objectivity, including interpretations based on expert opinions or previous literature may lead to conclusions influenced by prior assumptions.

Third, one might argue that other theories and models could have been considered to generate an explanatory intervention model proposal. However, given the large number of models, frameworks, or theories potentially to be considered for the generation of the explanatory intervention model, there was a need for a pragmatic decision regarding the number of models, frameworks, or theories to be taken forward [40, 92]. While the proposed model offers substantial contributions by expanding the usability of findings and integrating different factors, the limitations regarding subjectivity and the non-systematic nature of the review should be addressed in future research to enhance the rigor and reliability of the findings.

### Implications and future research directions

The findings from this scoping review offer valuable insights into the application of wearable technology for heat-exposed outdoor workers, particularly in the context of environmental health literacy [82] and precision prevention in occupational health [101]. The key question of context-mechanism-outcome configurations can be addressed by considering how such technology can enhance individual awareness, health behavior, and employer responsibility, particularly in high-risk outdoor environments. Real-time physiological and environmental monitoring can improve environmental health literacy, increase awareness of personal risk factors, such as heat stress, and enable timely preventive actions. This heightened awareness may contribute to better health outcomes as workers become more informed about how environmental conditions affect their well-being.

The use of wearable sensors aligns with the principles of precision prevention in occupational health [102] by tailoring interventions to individual workers’ needs and resources. By continuously monitoring physiological indicators (such as heart rate and body temperature) and environmental factors (such as ambient temperature), this technology allows for personalized preventive strategies. Workers can receive individualized feedback on their risk of heat-related illness, which can help them take appropriate preventive measures, such as adjusting their work intensity or hydrating.

Based on the findings, several practical recommendations can be delineated regarding the dissemination of information: Employers and workers should be made aware of the availability and usefulness of wearable-based technology for both physiological and environmental monitoring. Educational campaigns or targeted communications can help bridge knowledge gaps, ensuring that both parties understand the potential benefits of such technologies. Monitoring results from wearable devices need to be communicated understandably. This is essential to improve outdoor workers’ understanding and awareness of their individual heat exposure risk [55]. Simplified, user-friendly interfaces and regular feedback on heat exposure should be integrated into wearable systems to enhance workers’ ability to interpret and act on the data [36, 60, 61]. Outdoor workers should be equipped with wearable-based technology as part of their personal protective equipment [37]. Employers can incorporate these devices as a token of appreciation, reflecting a commitment to worker health and safety [49, 81]. This can foster a sense of value and responsibility, increasing worker engagement with preventive practices.

Further research and targeted actions will be essential to assess the usability of wearables and the acceptance regarding data privacy/ownership [36] while optimizing the deployment and effectiveness of these technologies. To further enhance the effectiveness of interventions, new study designs such as micro-randomized trials and just-in-time adaptive interventions should be considered. Micro-randomized trials allow for continuously adapting interventions in real time [103, 104]. By randomizing at the right decision-making time, micro-randomized trials can provide insights into the effectiveness of specific interventions (e.g., rest breaks or hydration prompts) under different conditions. This study design is particularly valuable for assessing how well wearable device-based interventions work in dynamic environments like outdoor workplaces, where heat exposure fluctuates throughout the day. Just-in-time adaptive interventions use a personalized, context-aware intervention model that delivers support exactly when needed. For outdoor workers, just-in-time adaptive interventions may provide timely notifications based on their physiological data (e.g., rising body temperature or heart rate) and environmental conditions (e.g., increased heat exposure). This approach ensures that interventions, such as reminders to hydrate or reduce physical activity, are provided at the right time, in the right place, to the right individual [105–107], they are most beneficial, minimizing the risk of heat stress for outdoor workers.

## Conclusions

Heat stress and strain are expected to become even more significant in the future due to the ongoing effects of climate change and increased exposure to extreme weather conditions. This scoping review provides a consolidated understanding of how wearable devices are currently used to support heat stress prevention among outdoor workers. It highlights that wearables are generally well-accepted and offer real-time, personalized monitoring of physiological and environmental parameters, which can inform timely health alerts and behavioral adaptations. The review identifies key contextual factors that influence the effectiveness of these interventions. Building on these insights, we developed an explanatory intervention model that integrates three established frameworks to guide the design and implementation of wearable-supported prevention strategies.

The model is designed to help identify what works, for whom, and in what circumstances [40], enabling the adaptation of current interventions and the development of new, more tailored strategies to protect outdoor workers. Integrating environmental health literacy into future research is essential, as it can empower outdoor workers to understand and act on the health risks posed by their work environment. By enhancing their awareness, knowledge, and motivation, thus health literacy, outdoor workers will be better equipped to take preventive actions, reducing their susceptibility to heat-related illness. Similarly, incorporating precision prevention in occupational health approaches [101] will ensure that interventions are customized to individual needs and conditions. Continuous monitoring of environmental and physiological parameters will allow for real-time, tailored feedback, leading to more effective heat stress prevention. Implementing and refining tailored interventions can minimize the risk of heat-related health issues, promoting long-term worker health and safety in increasingly challenging outdoor environments.

## Supplementary Information

Below is the link to the electronic supplementary material.

Supplementary Material 1

Supplementary Material 2

Supplementary Material 3

Supplementary Material 4

## Data Availability

All relevant data are included in the manuscript and its supporting information files.

## Abbreviations

°C: Degrees celsius
°F: Degrees fahrenheit
PRISMA-ScR: Preferred reporting items for systematic reviews and meta-analyses extension for scoping reviews
Wearables: Wearable technologies
WHO: World health organization
