# Supplementary material for: Wearable device-based interventions in heat-exposed outdoor workers — a scoping review and an explanatory intervention model
Source: BMC Public Health. 2025 Aug 22;25:2893. doi: 10.1186/s12889-025-24262-2 (PMC12372262; doi:10.1186/s12889-025-24262-2)
Supplement: Supplementary file 1 — Supplementary Material 1 [file 12889_2025_24262_MOESM1_ESM.docx]

Wearable device-based interventions

in heat-exposed outdoor workers – A scoping review and an explanatory intervention model

Julian Friedrich^1^*, Teresa S. Schick^1^, Filip Mess^1^, Simon Blaschke^1^*

^1^Technical University of Munich, TUM School of Medicine and Health, Department of Health and Sports Sciences, Munich, Germany

* Corresponding authors

E-mail: julian.friedrich@tum.de (JF), simon.blaschke@tum.de (SB)

**S1 File. Search strategy and term.**

*Search String employed in Web of Science*

((TI=(wearable* or "wearable sensor" or "sensor" or "sensor-based" or "wearable technology" or "wearable intervention" or "digital health monitoring" or "health

monitoring" or "physiological monitoring" or "environmental monitoring" or "behavioral

monitoring" or "psychological monitoring" or "digital intervention")) AND TI=("heat stress" or heat or "heat strain" or "heat illness" or temperature or "high temperature")) AND TI=(workplace or "work place" or worksite or "work site" or organisational or

organizational or occupational or "outdoor worker" or employee) or ((AB=(wearable* or "wearable sensor" or "sensor" or "sensor-based" or "wearable technology" or "wearable intervention" or "digital health monitoring" or "health monitoring" or "physiologic monitoring" or "environmental monitoring" or "behavioral monitoring" or "psychological monitoring" or "digital intervention")) AND AB=("heat stress" or heat or "heat strain" or "heat illness" or temperature or "high temperature")) AND AB=(workplace or "work place" or worksite or "work site" or organisational or organizational or occupational or "outdoor worker" or employee)

*Search String employed in PubMed*

((wearable*[Title/Abstract] OR "wearable sensor"[Title/Abstract] OR "sensor"[Title/Abstract] OR "sensor-based"[Title/Abstract] OR "wearable technology"[Title/Abstract] OR "wearable intervention"[Title/Abstract] OR "digital health

monitoring"[Title/Abstract] OR "health monitoring"[Title/Abstract] OR "physiologic

monitoring"[Title/Abstract] OR "environmental monitoring"[Title/Abstract] OR "behavioral monitoring"[Title/Abstract] OR "psychological monitoring"[Title/Abstract] OR "digital intervention"[Title/Abstract]) AND ("heat stress"[Title/Abstract] OR heat[Title/Abstract] OR "heat strain"[Title/Abstract] OR "heat illness"[Title/Abstract] OR

temperature[Title/Abstract] OR "high temperature"[Title/Abstract])) AND (workplace[Title/Abstract] OR "work place"[Title/Abstract] OR worksite[Title/Abstract]

OR "work site"[Title/Abstract] OR organisational[Title/Abstract] OR organizational[Title/Abstract] OR occupational[Title/Abstract] OR "outdoor worker"[Title/Abstract] OR employee[Title/Abstract])
