# Supplementary material for: Wearable device-based interventions in heat-exposed outdoor workers — a scoping review and an explanatory intervention model
Source: BMC Public Health. 2025 Aug 22;25:2893. doi: 10.1186/s12889-025-24262-2 (PMC12372262; doi:10.1186/s12889-025-24262-2)
Supplement: Supplementary file 2 — Supplementary Material 2 [file 12889_2025_24262_MOESM2_ESM.docx]

Wearable device-based interventions

in heat-exposed outdoor workers – A scoping review and an explanatory intervention model

Julian Friedrich^1^*, Teresa S. Schick^1^, Filip Mess^1^, Simon Blaschke^1^*

^1^Technical University of Munich, TUM School of Medicine and Health, Department of Health and Sports Sciences, Munich, Germany

* Corresponding authors

E-mail: julian.friedrich@tum.de (JF), simon.blaschke@tum.de (SB)

**S2 File. List of included studies.**

Al-Bouwarthan, M., Quinn, M. M., Kriebel, D., & Wegman, D. H. (2020). A Field Evaluation of Construction Workers’ Activity, Hydration Status, and Heat Strain in the Extreme Summer Heat of Saudi Arabia. *Annals of Work Exposures and Health*, *64*(5), 522–535. https://doi.org/10.1093/annweh/wxaa029

Culp, K., & Tonelli, S. (2019). Heat-Related Illness in Midwestern Hispanic Farmworkers: A Descriptive Analysis of Hydration Status and Reported Symptoms. *Workplace Health & Safety*, *67*(4), 168–178. https://doi.org/10.1177/2165079918813380

Hertzberg, V., Mac, V., Elon, L., Mutic, N., Mutic, A., Peterman, K., Tovar-Aguilar, J. A., Economos, E., Flocks, J., & McCauley, L. (2017). Novel Analytic Methods Needed for Real-Time Continuous Core Body Temperature Data. *Western Journal of Nursing Research*, *39*(1), 95–111. https://doi.org/10.1177/0193945916673058

Kakamu, T., Endo, S., Hidaka, T., Masuishi, Y., Kasuga, H., & Fukushima, T. (2021). Heat-related illness risk and associated personal and environmental factors of construction workers during work in summer. *Scientific Reports*, *11*(1), 1119. https://doi.org/10.1038/s41598-020-79876-w

Kakamu, T., Endo, S., Tsutsui, Y., Hidaka, T., Masuishi, Y., Kasuga, H., & Fukushima, T. (2022). Heart rate increase from rest as an early sign of heat-related illness risk in construction workers. *International Journal of Industrial Ergonomics*, *89*, 103282. https://doi.org/10.1016/j.ergon.2022.103282

Kim, A., & Yoo, G. (2023). Field-based Heatwave Risk Assessment of Outdoor Workers Measured by Wearable Sensors. *Asia-Pacific Journal of Atmospheric Sciences*, *59*(4), 447–458. https://doi.org/10.1007/s13143-023-00326-4

Kim, Y., Kim, J., Chicas, R., Xiuhtecutli, N., Matthews, J., Zavanelli, N., Kwon, S., Lee, S. H., Hertzberg, V. S., & Yeo, W. (2022). Soft Wireless Bioelectronics Designed for Real‐Time, Continuous Health Monitoring of Farmworkers. *Advanced Healthcare Materials*, *11*(13), 2200170. https://doi.org/10.1002/adhm.202200170

Mitchell, D. C., Castro, J., Armitage, T. L., Vega-Arroyo, A. J., Moyce, S. C., Tancredi, D. J., Bennett, D. H., Jones, J. H., Kjellstrom, T., & Schenker, M. B. (2017). Recruitment, Methods, and Descriptive Results of a Physiologic Assessment of Latino Farmworkers: The California Heat Illness Prevention Study. *Journal of Occupational & Environmental Medicine*, *59*(7), 649–658. https://doi.org/10.1097/JOM.0000000000000988

Pancardo, P., Acosta, F., Hernández-Nolasco, J., Wister, M., & López-de-Ipiña, D. (2015). Real-Time Personalized Monitoring to Estimate Occupational Heat Stress in Ambient Assisted Working. *Sensors*, *15*(7), 16956–16980. https://doi.org/10.3390/s150716956

Ruas, A. C., Maia, P. A., Roscani, R. C., Bitencourt, D. P., & Amorim, F. T. (2020). Heat stress monitoring based on heart rate measurements. *Revista Brasileira de Medicina Do Trabalho*, *18*(02), 232–240. https://doi.org/10.47626/1679-4435-2020-449

Runkle, J. D., Cui, C., Fuhrmann, C., Stevens, S., Del Pinal, J., & Sugg, M. M. (2019). Evaluation of wearable sensors for physiologic monitoring of individually experienced temperatures in outdoor workers in southeastern U.S. *Environment International*, *129*, 229–238. https://doi.org/10.1016/j.envint.2019.05.026

Shakerian, S., Habibnezhad, M., Ojha, A., Lee, G., Liu, Y., Jebelli, H., & Lee, S. (2021). Assessing occupational risk of heat stress at construction: A worker-centric wearable sensor-based approach. *Safety Science*, *142*, 105395. https://doi.org/10.1016/j.ssci.2021.105395

Sharma, M., Suri, N. M., & Kant, S. (2022). Analyzing occupational heat stress using sensor-based monitoring: A wearable approach with environmental ergonomics perspective. *International Journal of Environmental Science and Technology*, *19*(11), 11421–11434. https://doi.org/10.1007/s13762-021-03862-6

Spook, S. M., Koolhaas, W., Bültmann, U., & Brouwer, S. (2019). Implementing sensor technology applications for workplace health promotion: A needs assessment among workers with physically demanding work. *BMC Public Health*, *19*(1), 1100. https://doi.org/10.1186/s12889-019-7364-2

Sugg, M. M., Fuhrmann, C. M., & Runkle, J. D. (2018). Temporal and spatial variation in personal ambient temperatures for outdoor working populations in the southeastern USA. *International Journal of Biometeorology*, *62*(8), 1521–1534. https://doi.org/10.1007/s00484-018-1553-z

Sugg, M. M., Fuhrmann, C. M., & Runkle, J. D. (2020). Perceptions and experiences of outdoor occupational workers using digital devices for geospatial biometeorological monitoring. *International Journal of Biometeorology*, *64*(3), 471–483. https://doi.org/10.1007/s00484-019-01833-8

Togo, H., & Hirata, A. (2021). Novel Health Risk Alert System for Occupational Safety in Hot Environments. *IEEE Pulse*, *12*(4), 24–27. https://doi.org/10.1109/MPULS.2021.3094253

Uejio, C. K., Morano, L. H., Jung, J., Kintziger, K., Jagger, M., Chalmers, J., & Holmes, T. (2018). Occupational heat exposure among municipal workers. *International Archives of Occupational and Environmental Health*, *91*(6), 705–715. https://doi.org/10.1007/s00420-018-1318-3

Wang, S., Richardson, M. B., Wu, C. Y. H., Cholewa, C. D., Lungu, C. T., Zaitchik, B. F., & Gohlke, J. M. (2019). Estimating Occupational Heat Exposure From Personal Sampling of Public Works Employees in Birmingham, Alabama. *Journal of Occupational & Environmental Medicine*, *61*(6), 518–524. https://doi.org/10.1097/JOM.0000000000001604
