# Supplementary material for: Wearable device-based interventions in heat-exposed outdoor workers — a scoping review and an explanatory intervention model
Source: BMC Public Health. 2025 Aug 22;25:2893. doi: 10.1186/s12889-025-24262-2 (PMC12372262; doi:10.1186/s12889-025-24262-2)
Supplement: Supplementary file 4 — Supplementary Material 4 [file 12889_2025_24262_MOESM4_ESM.docx]

Wearable device-based interventions

in heat-exposed outdoor workers – A scoping review and an explanatory intervention model

Julian Friedrich^1^*, Teresa S. Schick^1^, Filip Mess^1^, Simon Blaschke^1^*

^1^Technical University of Munich, TUM School of Medicine and Health, Department of Health and Sports Sciences, Munich, Germany

* Corresponding authors

E-mail: julian.friedrich@tum.de (JF), simon.blaschke@tum.de (SB)

**S4 File. Quality appraisal of included studies based on relevance, richness, and rigor.**

| **Authors and years** | **Relevance** | **Richness** | **Rigor** |
| --- | --- | --- | --- |
| Al-Bouwarthan et al., 2020 (16) | High: directly measures heat strain, hydration, and activity in workers | High: detailed physiological data and context | Moderate: objective measures, limited causal inference |
| Culp & Tonelli, 2019 (49) | Moderate: focuses on heat-related symptoms in farmworkers | Moderate: self-reported symptoms and hydration | Low: descriptive with limited methodological detail |
| Hertzberg et al., 2017 (50) | Moderate: develops tools for temperature data analysis | Moderate: limited real-world intervention context | High: strong analytical framework |
| Kakamu et al., 2021 (58) | High: assesses heat illness risk in construction | High: environmental and personal factors included | High: systematic measures and analysis |
| Kakamu et al., 2022 (59) | High: early sign detection via heart rate | Moderate: focused on one biomarker | High: rigorous physiological monitoring |
| Kim, A. & Yoo, 2023 (63) | High: wearable sensors in practical settings | High: combined environmental and physiological data | Moderate: sound method, less generalizable |
| Kim, Y.-S. et al., 2022 (51) | High: real-time monitoring with wearables | High: application with context and data richness | High: integrated engineering and validation |
| Mitchell et al., 2017 (52) | High: evaluates physiological heat strain in farmworkers | High: detailed demographic, exposure, and physiological data | High: robust recruitment and protocol |
| Pancardo et al., 2015 (9) | Moderate: heat stress estimation model using wearables | Moderate: lacks deep user context or field deployment | Moderate: includes validation, but limited real-world application |
| Ruas et al., 2020 (61) | Moderate: heart rate monitoring in occupational settings | Moderate: physiological focus, limited context | Moderate: practical application, but lacks robust design |
| Runkle et al., 2019 (37) | High: evaluates wearable sensors in real outdoor conditions | High: rich environmental and physiological context | High: sensor comparison and application |
| Shakerian et al., 2021 (53) | High: focuses on construction site risk profiling | High: real-world task and environmental detail | High: robust technical integration |
| Sharma et al., 2022 (62) | High: analyzes occupational stress using environmental ergonomics | Moderate: environmental parameters included | Moderate: mostly descriptive, not interventional |
| Spook et al., 2019 (36) | High: assesses user perspectives and organizational context | High: valuable contextual data on implementation | High: thorough qualitative methodology |
| Sugg et al., 2018 (54) | High: evaluates personal ambient temperature variation | Moderate: less emphasis on subjective or behavioral factors | High: clear protocol and analysis |
| Sugg et al., 2020 (55) | High: explores perceptions of wearable use among workers | High: detailed behavioral and acceptance insights | High: rigorous qualitative analysis |
| Togo & Hirata, 2021 (60) | Moderate: heat alert system development | Moderate: focus on technology features rather than user context | Moderate: limited real-world testing |
| Uejio et al., 2018 (56) | High: occupational exposure across roles and settings | Moderate: descriptive exposure patterns | Moderate: generalizable, but not intervention-based |
| Wang et al., 2019 (57) | High: occupational heat exposure with personal sensors | Moderate: less focus on behavior or intervention mechanisms | High: robust environmental measurements |
